# Pilot of primary care physician discussion and resource allocation after screening for unintentional injuries and social determinants of health

**DOI:** 10.1186/s40621-019-0206-y

**Published:** 2019-05-29

**Authors:** Sarah Denny, Mike Gittelman, Hayley Southworth, Samantha Anzeljc, Melissa Wervey Arnold

**Affiliations:** 1 Division of Emergency Medicine, Nationwide Children’s Hospital, Columbus, 43205 USA; 2 Division of Emergency Medicine, Cincinnati Children’s Hospital Medical Center, Cincinnati, 45229 USA; 3 American Academy of Pediatrics, Ohio Chapter, Columbus, 43235 USA

**Keywords:** Injury prevention, Social determinants of health, Anticipatory guidance, Screening

## Abstract

**Background:**

Standardized screening tools used by pediatric providers can help determine a child’s injury and social risks. This study determined if an office-based quality improvement program could increase targeted anticipatory guidance and community resource distribution to families.

**Methods:**

Practices recruited from the Ohio Chapter, American Academy of Pediatrics’ database self-selected to participate in a quality improvement project. Two age-appropriate screening tools, corresponding talking points and local resources for birth–1 year and 1–5 year aged children were developed for unintentional injury and social health determinant topics. After a one-day learning session, practice teams implemented the tools into well-child care visits for children < 5 years of age. Two months of retrospective baseline data was collected for each participating clinician. During the 6-month collaborative, physicians randomly reviewed 5 screening tools monthly for each age category to identify injury and social risk discussions and to determine if resources were provided. Frequencies of counseling and resource distribution were calculated. Participating providers received Maintenance of Certification IV credit.

**Results:**

Ten practices (18 providers) participated and 667 tools (*n* = 313, birth-1 year, *n* = 354, 1–5 year) were collected. For birth–1 year, the most common risky behaviors were related to unintentional injuries: no CPR training 164(52%), car seat not checked 149(48%) and home furniture not secured 117 (37%). For 1–5 year screens, unintentional injuries were also most common: no CPR training 222(63%), car seat not checked 203(57%) and access to choking hazards 198(56%). Families practiced riskier behaviors for unintentional injuries compared to social risks for both age groups (birth – 1 year, social 189/4801 (4%) vs. unintentional injury questions 999/6260 (16%) and 1–5 years, social 271/5451 (5%) vs unintentional injury questions 1140/6372 (18%). From baseline, discussions increased from 31% to 83% for birth – 1 year and 24% to 86% for 1–5 year families. Resource distribution increased by 63% for birth-1 year and 69% for 1–5 year families by pilot conclusion.

**Conclusions:**

Using standardized screening tools in an office setting shows that families often practice unintentional injury risks more than having social concerns. After screening, appropriate resources can be provided to families to encourage behavior change.

## Background

Injury continues to be the leading cause of death and disability for US children (Vital signs, 2012); costing the healthcare system more than $81 billion per year (Miller et al., 2000). Researchers in the field of injury prevention continue to search for effective ways to decrease this epidemic. Anticipatory guidance regarding injury prevention (IP), an approach to reduce injuries, is recommended by the American Academy of Pediatrics (AAP) Bright Futures (Hagan et al., 2007) and other professional organizations (Statement on Firearm Injuries, 2013; Injury and Violence Prevention, 2020); however, many factors, including lack of time and training, preclude providers from having these IP discussions (Yarnall et al., 2003; Wright, 1997; Belamarich et al., 2006). Despite a lack of pediatric care providers (PCPs) counseling about unintentional injury prevention practices, recent literature has shown this practice can be effective in changing family behavior (Zonfrillo et al., 2018). In particular, risks such as falls, poisonings, burns/fire, traffic and drowning have been shown to have positive effects after physician counseling (Zonfrillo et al., 2018).

In addition to unintentional injury risks, pediatric providers are also obligated to screen for intentional injury and social risks in the home setting. The Safe Environment for Every Kid (SEEK) model was developed to help pediatric care providers identify and address targeted risk factors for child maltreatment and social concerns of families with young children (Dubowitz, 2014).

Approximately 1546 children died in the United States from child abuse and neglect in 2014 (DataBank, C.T, 2016). Recent literature indicates that risk assessments and behavioral interventions in pediatric clinics reduce abuse and neglect outcomes (Nelson et al., 2013). Also, researchers have found that children of unemployed parents and lower socioeconomic status are at greater risk of premature death (Edwards et al., 2006; Cubbin & Smith, 2002). High risk clinics who participated in the SEEK program had fewer Child Protective Services reports, fewer instances of medical neglect, or noncompliance, fewer children with delayed immunizations and fewer instances of severe physical assault (Dubowitz, 2014). Additionally, the SEEK tool did not require any additional provider time and it was estimated that approximately $37 million would be saved if the SEEK model was provided to 100,000 families (Dubowitz, 2014).

Although past literature clearly supports that tailored screening by PCPs, with individualized, custom messaging, helps to address specific needs, changes behaviors, and saves health care dollars, this approach is not used consistently by PCP offices (Nansel et al., 2002; Gittelman et al., 2018). By implementing standardized tools into pediatric offices using Quality Improvement (QI) methodology, PCPs are able to screen for and discuss high-risk injury and social topics in a more pertinent and efficient manner (Gittelman et al., 2015). The purpose of this pilot QI study was to provide PCP offices with a standardized screening tool that addresses age-based injury prevention and social determinants of health (SDH) issues, and determine if providers increased their screening, targeted counseling and resource distribution for these screened issues.

## Methods

### Study design

The Ohio Chapter, American Academy of Pediatrics (OAAP) conducted two similar waves of a Quality Improvement Learning Collaborative (QILC) to improve screening and counseling about IP and SDHs along with appropriate resource distribution at well child visits (WCV). The data for this study comes from the two waves conducted from July 13, 2017 to December 14, 2017 and from January 30, 2018 to July 30, 2018. The aim of this program was to understand the most common IP and SDH risks families with children < 5 years of age encounter and determine if PCPs increase counseling and appropriately provide families local resources when indicated.

#### Tool development and resource provided

A hybrid tool to screen for high-risk behaviors, using a previously established IP screening tool (Gittelman et al., 2018) as well as the SEEK screening tool for SDH risks was created (tool available upon request). A test-retest study has shown the injury tool has good reliability (Gittelman et al., 2018). Age-appropriate unintentional injury prevention questions were developed for each age specific category (birth-1 year and 1–5 years). The SEEK team gave consent for us to utilize their tool in this format. Topics covered in both the birth to 1 year tool and the 1–5 year tool are listed in Table 1. Some topics had more than one question per topic (eg. child passenger safety) in order to cover the most up-to-date and relevant prevention information. The screening tool, answer sheet, and resource sheet were all on paper to accommodate practices not yet on an electronic medical record.

**Table 1:** Age-based Topics Covered in Screening Tool

Category Covered	Topic Covered	Birth to 1 year (37 questions)	1–5 years (35 questions)
SEEK			
	Employment	X	X
	Child care	X	X
	Utilities	X	X
	Substance abuse	X	X
	Access to food	X	X
	Maternal depression	X	X
	Concerns for child’s safety	X	X
	Concerns for interpersonal violence	X	X
Unintentional Injury			
	Furniture safety	X	X
	Falls	X	X
	Child passenger safety	X	X
	Safe sleep	X	X
	Poison prevention	X	X
	Firearm safety	X	X
	CPR knowledge	X	X
	Foreign body prevention	X	X
	Water safety	X	X
	Fire safety	X	X
	Burn prevention	X	X

In addition to age-appropriate screening questions, the tool also had a column for an individual in the office staff to check whether the caregiver’s response was appropriate or if counseling was recommended. Another column on the tool listed if discussion by the provider was completed. Healthcare providers were provided talking points for each topic area covered on the Injury Prevention + SEEK (IP + SEEK) tool, as well as an answer key for easy assessment.

Customized resource sheets were provided to each practice for distribution to families. Local and national resources were provided for each topic area covered by the screening tool. These local resources were obtained by reviewing online materials and making confirmation calls, as needed, for each screening tool topic in each county of participating PCP offices. Resource information provided included phone numbers and websites to local and national resources, as well as basic age-appropriate information on each topic. If discussion about a topic was recommended, resource allocation was to be provided to the caregiver.

#### Performance measures

The QILC performance measure percentages for screening, documentation and resource allocation were chosen based on previous QI injury prevention study work and they were increased to promote greatest change by the practice (Gittelman et al., 2015).

The two main performance measures for this QI program included:

1. At all WCVs, PCPs will cover at least 90% of the recommended age appropriate IP + SEEK counseling for that WCV. (Covered means families answered the screening question appropriately or when answered as high risk, PCPs discussed the high risk response)
2. Resources will be provided to families > 75% of time when indicated.

### Setting and QI program

The QILC structure was similar to the Institute of Healthcare Improvement (IHI) Breakthrough Series Collaborative (The Breakthrough Series: IHI’s Collaborative Model for Achieving Breakthrough Improvement, 2003). Practitioners were recruited for both waves of each collaborative from the OAAP membership database and volunteered to participate. A focus was placed on recruiting practices serving a higher than average number of Medicaid recipient families. A goal of reaching at least 20 total pediatric healthcare providers across two waves was set. Table 2 outlines the demographic information of the recruited participating practices and providers. Core teams from each participating practice were chosen and consisted of: a physician leader, a nurse/nurse practitioner or medical assistant and an administrative staff/office manager. Core teams participated in a pre-work conference call outlining the requirements for the QILC and the collection of baseline data.

**Table 2:** Demographic information of the participating practices

Practice	Estimated Annual Patient Volume	% Self Pay	% Private Insurance	% Medicaid	Location Type (Urban/Rural)	Number of Participants
1	1000	10	60	30	Urban	3
2	4000	0	0	100	Urban	1
3	1500	10	70	20	Rural	1
4	500	1	49	50	Urban	1
5	XXX^a^	XXX^a^	XXX^a^		Suburban	1
6	680	3	11	86	Urban	1
7	600	5	90	5	Suburban	4
8	300	10	50	40	Urban	2
9	1000	0	40	60	Rural	3
10	2600	2	52	46	Suburban	1

^a^ data not available

At least one core team member from each practice attended a one-day, face-to-face learning session held July 13, 2017 for Wave 1 or January 30, 2018 for Wave 2, with a webinar option for those who could not attend in person. During the learning session, teams were taught about the importance of discussing IP at a WCV, principles of QI methodology, including how to conduct Plan-Do-Study-Act (PDSA) cycles, how to implement the IP + SEEK screening tool into practice, review of the resources provided, and how monthly data should be collected and reported. At the conclusion of the learning session, each team was provided with screening tools, grading sheets, physician talking points, customized resource sheets and contact information.

During the QILC, PCPs tried to address all risky behaviors elicited from families and provide necessary resources. Each month, core team members participated in a webinar to review practice team and collaborative data so that areas of success or needs for improvement could be addressed. The action period calls consisted of a 15-min lecture on a topic relevant to IP, SDH or QI concepts, followed by review of the data. Finally, each team had to submit a total of three PDSA worksheets demonstrating tests of change over the course of the learning collaborative.

All physician members in each practice that submitted data received American Board of Pediatrics (ABP) Maintenance of Certification (MOC) IV credit for participation as well as sleep sacks, board books, and cabinet locks for distribution within their practice.

### Data collection

#### Baseline/pre-work

Participating pediatric providers reviewed 12 randomly selected charts (6 for each WCV group: 0–1 year and 1–5 years) from the previous two months of WCVs prior to the QILC. A standardized protocol for chart review was provided to pediatric providers prior to the learning session and reviews were entered into SurveyMonkey®. Baseline data abstracted from charts consisted of whether providers counseled on each of the IP + SEEK screening tool topics; use of resources and referrals were not collected. Practices reviewed their baseline data at the learning session.

#### Action period

After the learning session, pediatric providers randomly selected 5 IP + SEEK screening tools for each WCV age range (birth-1 year or 1–5 years) and entered tools into SurveyMonkey®. Topics were considered addressed if the family answered the screening question appropriately (based on the provided answer key) or if the provider discussed the topics with high risk responses (indicated by provider checking the discussed box on the tool). Frequencies were determined to assess all high risk responses to age appropriate topics addressed by the PCP at WCVs, and if resources were provided. Changes in providers addressing risky topics and providing resources over time were determined and presented individually and in aggregate on the action period calls in data charts, used to hone improvement efforts.

### Analysis

Data from each wave of the QILC were combined and aggregate frequencies were calculated each month during the QILC using Microsoft Excel Office 365®. Data charts displayed frequencies for providers’ counseling that addressed topics with families’ high risk responses to questions and whether resources were provided to a family when a risky behavior was identified. Charts showing changes by practices over time were developed to show change during the collaborative. Separate frequencies were calculated for inappropriate responses and discussions for SEEK and IP risks and potential differences were evaluated using a chi square test using Microsoft Excel Office 365®.

### Human subjects review

Approval was obtained from the Cincinnati Children’s Hospital Institutional Review Board prior to study initiation.

## Results

Ten practices (18 providers) participated in the collaborative. Six of the practices (60%) only had one of their providers participate, and six (60%) cared for a Medicaid population > 40% of the time. Practices varied in size having an annual patient volume between 300 and 4000 patients (Table 2).

Sixty-eight charts for birth-1 year and 101 charts for WCV 1–5 years were reviewed at baseline. Documentation of addressing IP + SEEK topics occurred 31% of the time for families of newborn to 1-year old and 24% of the time for families with children 1 to 5 years of age prior to implementing the IP + SEEK tool. At baseline, the most common topics addressed for birth to 1 year included: placing the child to sleep on their back (82%), placing car seats in the back seat, facing backward (75%), and placing the child to sleep in their own sleeping space (74%). The common risks addressed for children 1–5 years old were counseling caregivers who feel that their child (ren) are difficult to take care of (64%), maternal depression (58%) and use of a car safety seat in the car for every trip (56%).

Data from 313 IP + SEEK screening tools were submitted from birth-1 year, and 354 were submitted from WCV 1–5 years during the action period. On average, families reported at least 4–5 risks on each screening tool for birth − 1 year and 1–5 years. The most common risky behaviors for birth–1 year were unintentional injury topics: no CPR training 164(52%), car seat not checked 149(48%), and home furniture not secured 117 (37%). For the 1–5 years IP + SEEK screens, unintentional injuries were also most common: no CPR training 222(63%), car seat not checked 203(57%), and access to choking hazards 198(56%).

**Fig. 1:**
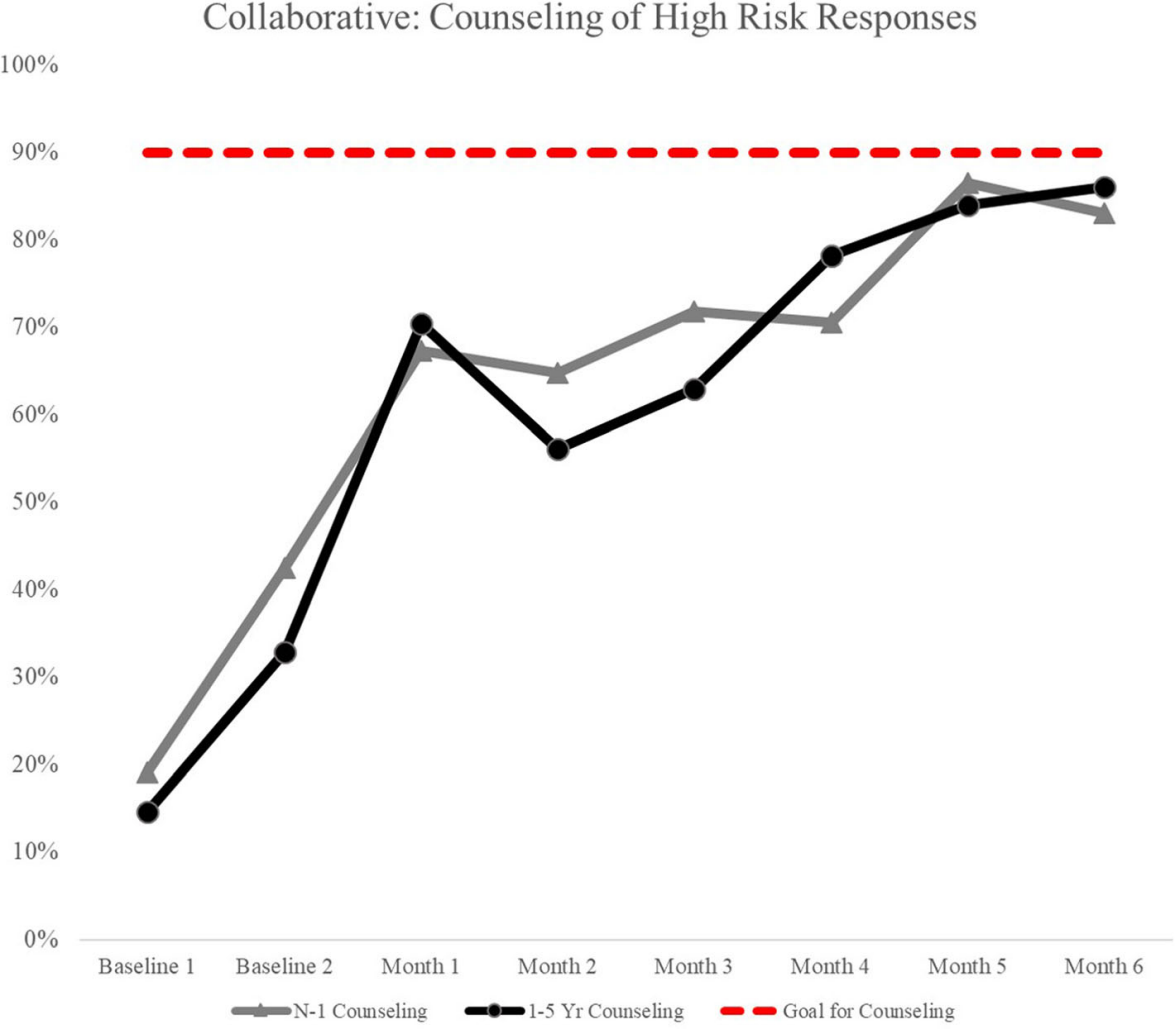
Counseling of High Risk Responses

During the collaborative, counseling from baseline on risky topics improved by 52% for birth-1 year and by 62% for 1–5 years tools. By the end of the collaborative, questions with high risk responses were addressed 83% of the time for birth-1 year and 86% of the time for 1–5 years screening tools (Fig. 1). In the first month of the collaborative, providers offered resources 65% of the time for each WCV age group. Provision of resources peaked in month four of the collaborative for the 1–5 years age group, with 82% of families receiving a resource and peaked in month five for WCVs birth-1 year, with 79% of families being offered a resource when indicated. By the sixth and final month of the collaborative, 63% of birth-1 year and 69% of 1–5 years families were receiving a resource when questions had a high risk response.

IP + SEEK screening tool topics were stratified by social and unintentional injury topics. There were more unintentional injury related questions answered with high risk responses than social questions for both age categories: birth – 1 year, social 189/4801 (4%) vs. unintentional injury questions 999/6260 (16%) and 1–5 years, social 271/5451 (5%) vs. unintentional injury questions 1140/6372 (18%) (Table 3). There is a statistically significant difference between the number of injury questions answered as high risk and the number of social determinant questions answered as high risk for both age ranges (birth-1 year tool χ^2^ (1) = 409.61, *p* < 0.001; 1–5 years tool χ^2^ (1) = 466.54, p < 0.001).

**Table 3:** Frequencies and statistical significance between unintentional injury and social answers

Observed Frequencies				Expected Frequencies				
	Social	Unintentional	Total	Social	Unintentional			
Birth – 1 Year								
High Risk Answers	189	999	1188	515.65	672.35	Chi-square	Degrees of freedom	*p*-value
Correct Answers	4612	5261	9873	4285.35	5587.65	409.61	1	*p* < 0.00001
Total Answers	4801	6260	11,061					
1–5 Years								
High Risk Answers	271	1140	1411	650.54	760.46	Chi-square	Degrees of freedom	*p*-value
Correct Answers	5180	5232	10,412	4800.46	5611.54	466.54	1	*p* < 0.00001
Total Answers	5451	6372	11,823					

**Fig. 2:**
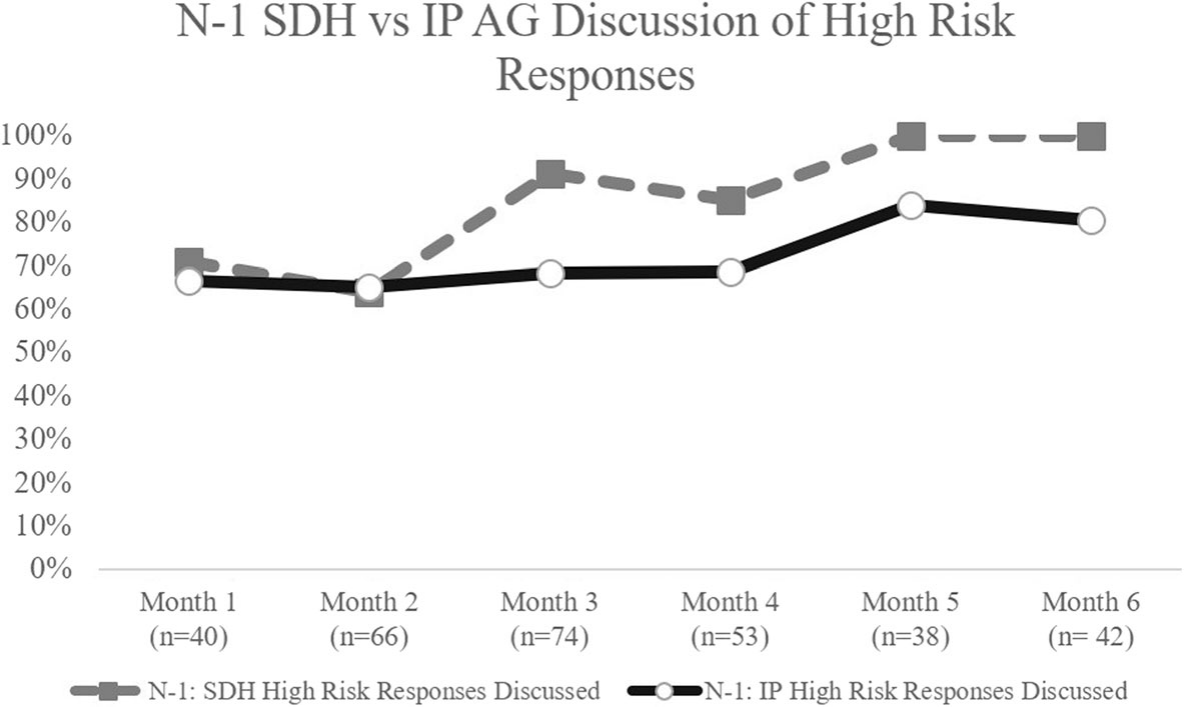
Newborn to 1 Year Old Social Determinants of Health vs. Injury Prevention Anticipatory Guidance Discussion of High Risk Responses

**Fig. 3:**
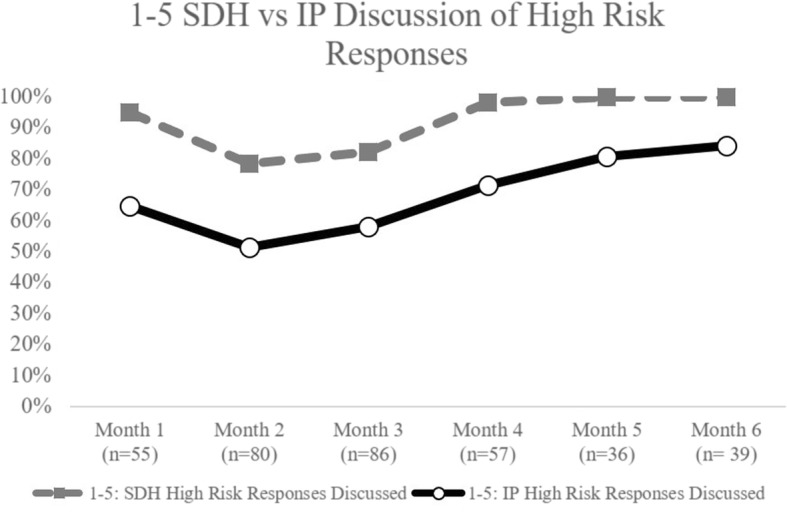
One to Five Year old Social Determinants of Health vs Injury Prevention anticipatory Guidance Discussion of High Risk Responses

Over the course of the action period, providers increasingly addressed SEEK and IP topics that had high risk responses. On average, 46% of birth-1 year tools had high risk responses on SEEK questions, whereas 90% of tools for this age had high risk responses for IP questions. During the QILC, for birth - 1 year visits, counseling about high risk SEEK question responses improved from 71% to 100%, while counseling on IP questions only improved from 67% to 80% by end of the collaborative (Fig. 2). Approximately 47% of 1–5 years tools had high risk responses on SEEK questions and 94% had high risk responses on IP questions. There was a 5% increase (95% to 100%) in the number of tools that had all high risk SEEK question responses addressed by the provider (Fig. 3). A more notable increase of 19% (65% to 84%) occurred for addressing all IP questions addressed in this age range.

## Discussion

This study demonstrates several important findings. We determined that healthcare providers in the primary care setting can implement a screening tool to evaluate for IP and SDH risks into WCVs. Using the IP + SEEK tool increased screening, topic discussion and resource distribution from baseline. Families with children in both age categories tend to report more unintentional injury risks than social risks for our population.

Similar to previous studies that introduced an IP screening tool into pediatric WCVs, we showed that providers can improve their counseling of risky behaviors over time (Gittelman et al., 2015). In just 6 months, looking at children < 1 year of age, counseling about risky behaviors improved to 80%. Also, as in this previous study, similar risky behaviors were elicited, including no CPR training, car seat inspection, and furniture being secured to the walls for children < 1 year old (Gittelman et al., 2015). This finding is likely because these prevention techniques require a greater effort by the family (e.g. going to a training or securing an item to the wall) as opposed to placing the poison center number close to a telephone. Securing furniture presents specific challenges, depending on the type of housing. Those families living in a rental property or apartment might be more hesitant to secure anything to the wall. Although furniture tip overs cause a pediatric death every 2 weeks (Suchy, 2016), healthcare providers and families may not be aware of this significant injury risk.

Interestingly, respondents were more likely to respond incorrectly to IP than SEEK questions. However, healthcare providers were more likely to address high risk SEEK compared to IP questions. We believe that a lack of training about IP (Wright, 1997) or the increased focus on the role SDH in the long term health of children and adults played a role in this difference of counseling (Foley et al., 2018; Yaeger et al., 2018; Conroy et al., 2010). Further study is needed to determine why families are more likely to answer IP questions incorrectly than the SDH questions.

In previous QILCs, participants reported that having a customized resource sheet would be helpful when counseling patients on IP topics (Gittelman et al., 2015). Having a resource sheet with both local and national organizations, tailored specifically for each practice made it easy for healthcare providers to link families with resources that fit their needs. The resource sheet that was provided included both SEEK and IP resources, so even if a family did not document a SEEK need, they were given the resources, in case a need was either not disclosed, or a need developed at a later date. Putting all the resources on 1 double-sided page streamlined the process for providers and office staff.

Sustainability can be a challenge in QI projects. While we have not collected data following the completion of this IP + SEEK QILC, we did discuss sustainability on action period calls and had providers think about how they would continue to incorporate the screening tool and resource sheet in their ongoing practice. An informal survey of providers who participated in previous waves indicates that 60% of respondents were still using portions of the tool or resources sheet 6–18 months after completion of the project.

We did not have balancing measures included as a part of our measures and change package; however, providers were informally queried on Action Period Conference Calls, One-on-One team practice coaching calls and in an exit interview on potential unintended consequences of using the screening tool. Visit length or changes in topics previously addressed not on the screening tool were not specifically tracked. While providers noted that length of time with families who needed discussion on many topics was a barrier, many also shared that having a focused list of topics for discussion, based upon a graded screening tool, improved efficiency of visits overall.

There are several limitations to this study. There is a selection bias since the providers sought out the opportunity to participate in this IP + SEEK QILC. As a result, providers may be more likely to instigate change in their practice since they were motivated to participate. Additionally, because providers were those more engaged with the OAAP and self-selected to participate, they may already be aware of QI practices and they were readier to implement screening tools into practice on a regular basis. Participating providers were asked to randomly select 5 charts per age group each month and report the data. A potential selection bias could have occurred as providers may have not consistently used the provided tools and only counseled for these 5 patients of each age category monthly. On action period calls, we tried to eliminate this possibility by having discussions with participants. Social desirability bias could also affect answers to the SEEK questions causing families to have less incorrect responses on SEEK topics. Lastly, since this project was QI, as opposed to a randomized controlled trial, the efficacy of the screening tool is not definitive and warrants additional study.

## Conclusion

Primary care providers can implement a standardized screening tool that addresses age-based IP and SDH issues into the WCV. Providers increased counseling about injury behaviors and SDH topics when families self-identified risks. Unintentional IP risks tended to be more prominent than SDH concerns and thus screening for IP should be emphasized for all families with children < 5 years of age at WCVs.

## Abbreviations

AAP: American Academy of Pediatrics
ABP: American Board of Pediatrics
IHI: Institute of Healthcare Improvement
IP + SEEK: Injury Prevention + Safe Environment for Every Kid
IP: Injury prevention
MOC: Maintenance of certification
OAAP: Ohio Chapter, American Academy of Pediatrics
PCP: Pediatric care provider
PDSA: Plan-Do-Study-Act
QI: Quality improvement
QILC: Quality improvement learning collaborative
SDH: Social determinants of health
SEEK™: Safe environment for every kid
WCV: Well child visit
